# Incidence, management and outcome of women requiring massive transfusion after childbirth in the Netherlands: secondary analysis of a nationwide cohort study between 2004 and 2006

**DOI:** 10.1186/s12884-017-1384-7

**Published:** 2017-06-19

**Authors:** Paul I. Ramler, Thomas van den Akker, Dacia D. C. A. Henriquez, Joost J. Zwart, Jos van Roosmalen

**Affiliations:** 1 Department of Obstetrics, Leiden University Medical Center, Leiden, the Netherlands; 2 Department of Obstetrics and Gynecology, Haaglanden Medical Center, The Hague, the Netherlands; 3 Department of Obstetrics and Gynecology, Haga Hospital, The Hague, the Netherlands; 4 Department of Obstetrics and Gynecology, Deventer Hospital, Deventer, the Netherlands; 5 Athena Institute, VU University, Amsterdam, the Netherlands

**Keywords:** Postpartum hemorrhage, Blood transfusion, Obstetrics, Maternal mortality, Maternal morbidity

## Abstract

**Background:**

Postpartum hemorrhage remains the leading cause of maternal morbidity and mortality worldwide. Few population-based studies have examined the epidemiology of massive transfusion for postpartum hemorrhage. The aim of this study was to determine the incidence, management, and outcomes of women with postpartum hemorrhage who required massive transfusion in the Netherlands between 2004 and 2006.

**Methods:**

Data for all women from a gestational age of 20 weeks onwards who had postpartum hemorrhage requiring eight or more red blood cell concentrates were obtained from a nationwide population-based cohort study including all 98 hospitals with a maternity unit in the Netherlands.

**Results:**

Three hundred twenty-seven women who had postpartum hemorrhage requiring massive transfusion were identified (massive transfusion rate 91 per 100,000 deliveries (95% confidence interval: 81–101)). The median blood loss was 4500 mL (interquartile range 3250–6000 mL) and the median number of red blood cell concentrates transfused was 11 units (interquartile range 9–16 units). Among women receiving massive transfusion, the most common cause of hemorrhage was uterine atony. Eighty-three women (25%) underwent hysterectomy, 227 (69%) were admitted to an intensive care unit, and three women died (case fatality rate 0,9%).

**Conclusion:**

The number of women in the Netherlands who had postpartum hemorrhage treated with massive transfusion was relatively high compared to other comparable settings. Evidence-based uniform management guidelines are necessary.

**Electronic supplementary material:**

The online version of this article (doi:10.1186/s12884-017-1384-7) contains supplementary material, which is available to authorized users.

## Background

Around the globe, postpartum hemorrhage (PPH) continues to be a leading cause of both maternal morbidity and mortality [1, 2]. In the Netherlands, PPH is defined by at least 1000 mL blood loss within 24 h of giving birth. There has been an increase in the incidence of PPH among all births in the Netherlands from 4.1% in 2000 to 6.4% in 2013 [3]. Although case definitions of PPH vary between countries, this rising incidence of PPH is also evident in other high-income countries [4–8]. An explanation for the increasing rates of PPH remains unclear.

In high-income countries, pharmacological, mechanical and surgical methods as well as radiological arterial embolization are available to arrest heavy bleeding. In case of life-threatening PPH, access to and use of sufficient quantities of blood products for transfusion to treat severe anemia and correct clotting disorder are critical [9, 10]. Clinical benefit of blood transfusion in obstetric hemorrhage was demonstrated in a hypothetical experimental study showing a 6.5-fold increase in risk of maternal death had red blood cell concentrates (RBCs) not been available, as is the case in many low-income settings [11].

Little is known about the management and outcomes of women who sustain PPH requiring massive transfusion. A recent population-based study from the United Kingdom (UK) indicated that PPH requiring massive transfusion was associated with high rates of morbidity and hysterectomy [12]. While the rate of obstetric transfusion in the Netherlands has decreased dramatically over time (from 23% in 2000 to 3.9% in 2013), transfusion rates in other high-income countries increased [3, 13, 14]. If rates of massive transfusion in the Netherlands were to have decreased over time, these data would be important for informing discussion about best transfusion practices for PPH management. The objective of our present study was to determine incidence, causes, management and clinical outcome of women with PPH requiring massive transfusion in the Netherlands.

## Methods

To determine incidence, causes, management approaches, and clinical outcomes of women who had PPH requiring massive transfusion in the Netherlands, we performed a secondary analysis of data from the LEMMoN study (Landelijke studie naar Etnische determinanten van Maternale Morbiditeit in Nederland) [15].

The LEMMoN study included 358,874 deliveries with severe acute maternal morbidity that occurred in 98 hospitals with a maternity unit in the Netherlands (100%) between 1st August 2004 and 1st August 2006. Severe acute maternal morbidity was categorized into five groups: intensive care unit (ICU) admission, uterine rupture, eclampsia/HELLP syndrome, major obstetric hemorrhage and a miscellaneous group. Major obstetric hemorrhage was defined as a need for transfusion of four or more units of erythrocyte concentrate or embolization or hysterectomy following PPH. Detailed information about study design and data collection were described previously [15]. For this specific study, we selected women from the LEMMoN cohort who were classified as ‘major obstetric hemorrhage’, had a gestational age of at least 20 weeks and received massive transfusion, defined as eight or more red blood cell concentrates within the first 24 h after delivery.

We recorded maternal characteristics (age, body mass index (BMI), geographical ethnic origin (the Netherlands, other European Countries or non-Western immigrants, subdivided into Middle East/North Africa, sub-Saharan Africa, South America and Far East), parity, previous experienced PPH and previous cesarean section), pregnancy characteristics (gestational age, mode of delivery, induction of labour, multiple pregnancy and preeclampsia) and specific data on postpartum hemorrhage (amount of blood loss, number of red blood cell concentrates transfused, hemoglobin levels at onset of bleeding, after bleeding and at day of discharge). Maternal and pregnancy characteristics of women in this study were compared to the general pregnant population of the Netherlands, obtained from Statistics Netherlands (CBS) and National Perinatal Database (LVR-2). Incidence figures in LVR-2 were multiplied by 59/100 to also represent all deliveries under primary care (41% in 2002) [16, 17].

Since postpartum hemorrhage is often the result of concurrent causes, we re-examined all cases of massive transfusion within the LEMMoN-cohort and registered up to three causes of PPH requiring massive transfusion for each individual case. Only those causes that contributed significantly to the hemorrhage were registered. These causes were noted as uterine atony, uterine rupture, iatrogenic during/after cesarean section, placental abnormalities (including retained placenta, placental remnant, placenta previa, abnormally invasive placenta and placental abruption (resulting in continuous hemorrhage in the postpartum period), laceration of vagina and/or cervix, primary clotting disorder with or without amniotic fluid embolism, rupture of the uterine artery, rupture of the liver capsule and uterine inversion. Causes of hemorrhage were analyzed by mode of birth (spontaneous vaginal delivery, instrumental vaginal delivery, elective cesarean section, emergency cesarean section and termination of pregnancy) and number of red blood cell concentrates transfused (‘moderate’ (8–12 units), ‘high’ (13–20 units) and ‘immense’ (>20 units). The cut-off points for the number of red blood cell concentrates transfused were identical to those described by Green et al. [12].

Management of postpartum hemorrhage requiring massive transfusion was divided into uterotonic agents (oxytocin, prostaglandin F2α, ergometrine, misoprostol), non-uterotonic drugs (tranexamic acid), mechanical interventions (intrauterine balloon, intrauterine packing and intra-abdominal packing), surgical interventions (removal of the placenta not performed during cesarean section, laparotomy, re-laparotomy, B-Lynch suture, uterine artery ligation and hysterectomy) and uterine artery embolization. Outcome of women was determined by need for hysterectomy, length of hospitalization, ICU admission, morbidity, maternal deaths and case fatality rate.

Statistical analyses were performed using IBM SPSS Statistics (version 22.0; SPSS Inc., Chicago, IL). Discrete data were summarized as frequencies and percentages, while continuous data were noted as medians with an interquartile range (IQR) expressed as the 25th and 75th percentiles. Women with a missing value for a specific parameter were excluded when calculating the rate for that variable.

## Results

During the study period, 358,874 deliveries took place in the Netherlands; 336 women sustained PPH and were given eight or more units of red blood cell concentrates. Of these women, nine were excluded due to a gestational age below 20 weeks, leaving 327 women for analysis. Incidence of massive transfusion due to PPH was 91 per 100,000 deliveries (95% confidence interval [95% CI]: 81–101). Clinical and demographic baseline characteristics for women are presented in Table 1 and characteristics of the pregnancies in Table 2. The median (IQR) age, BMI and gestational age were 33 years (30–36 years), 23 kg/m^2^ (21–26 kg/m^2^) and 38 weeks (37–41 weeks), respectively.

**Table 1:** Characteristics of the women

	N	(%)	General pregnant population in the Netherlands (%)^a^
Age (years)			
20–34	208	(63)	(75.3)
35–39	94	(29)	(21.3)
≥ 40	25	(8)	(3.4)
BMI (kg/m^2^)			
< 18,5	15	(5)	(3.1)
18,5–24,9	137	(42)	(65.2)
25,0–29,9	39	(12)	(21.9)
≥ 30	24	(7)	(9.8)
Unknown	112	(34)	–
Geographical ethnic origin			
The Netherlands	223	(68)	N/A
Other European Countries	7	(2)	N/A
Non-Western immigrants;	70	(22)	(16.8)
Middle East/North Africa	28	(7)	N/A
Sub-Saharan Africa	17	(5)	N/A
South America	16	(5)	N/A
Far East	9	(3)	N/A
Unknown	27	(8)	–
Parity			
0	158	(48.3)	(45.2)
1–2	145	(44.3)	(49.8)
≥ 3	24	(7.3)	(5.0)
Previous postpartum hemorrhage	40	(12)	N/A
Previous cesarean section	66	(20)	(6.0)

*N/A* data not available

^a^National reference values from Statistics Netherlands (exact study period) [16]

**Table 2:** Characteristics of pregnancy and birth

	N	(%)	General pregnant population in the Netherlands (%)
Gestational age			
Preterm (<37 weeks);	86	(26)	(5.8)^b^
20–24 weeks	6	(2)	N/A
24–32 weeks	18	(5)	N/A
32–37 weeks	62	(19)	N/A
Full Term	241	(74)	(94.2)^b^
Mode of delivery ^c^			
Vaginal	131	(40)	(78.4)^b^
Instrumental	43	(13)	(8.6)^b^
Cesarean Section;	151	(46)	(13.0)^b^
Elective	46	(14)	N/A
Emergency	105	(32)	N/A
Induction of labour	100	(31)	(12.5)^b^
Multiple pregnancy	37	(11)	(1.7)^a^
Preeclampsia during pregnancy	54	(17)	(4) [19]

*N/A* data not available

^a^National reference values from Statistics Netherlands (exact study period) [16]

^b^National reference values from the Netherlands Perinatal Registry (LVR-2, 2005) [17]

^c^In case of multiple births were the mode of delivery differed between the neonates, the mode of delivery refers to the most invasive mode

### Characteristics of postpartum hemorrhage requiring massive transfusion

The median (IQR) estimated blood loss was 4500 mL (3250–6000 mL), resulting in a median (IQR) hemoglobin drop from 11.6 g/dL (10.8–12.41 g/dL; data missing for 60 women) before hemorrhage to 5.96 g/dL (5–6.77 g/dL; data missing for 34 women) after hemorrhage. The median (IQR, range) number of red blood cell concentrates transfused was 11 (9–16, 8–52) (see Additional file 1 for distribution).

The most common cause of PPH requiring massive transfusion was uterine atony, followed by retained placenta and placenta previa (Table 3). For 117 women (36%), two causes were registered and for 12 women (4%) three. The commonest combinations for women with two causes were uterine atony with retained placenta (*N* = 28), uterine atony with placental remnant (*N* = 21) and uterine atony with cervical laceration (*N* = 10). For women with three causes, the most frequent combination was uterine atony with placental remnant and cervical laceration (*N* = 3). For nine women, no cause could be established. The 22 causes in the ‘other’ category in Table 3 were primary clotting disorder without amniotic fluid embolism (*N* = 7), uterine artery rupture (*N* = 6), live capsule rupture (*N* = 4), clotting disorder due to amniotic fluid embolism (*N* = 4) and uterine inversion (*N* = 1). Massive transfusion occurred during normal working hours (between 0800 and 1600 on a weekday) for 196 (65%) women; data were missing for 25 women. The onset of hemorrhage occurred at home for 52 (16%) women; data were missing for 7 women.

**Table 3:** Causes of PPH cases requiring massive transfusion^a^

	N	(%)
Uterine atony	179	(55)
Placenta abnormalities;	173	(53)
Retained	54	(17)
Previa	37	(11)
Abnormally invasive placenta	32	(10)
Remnant	30	(9)
Abruption	20	(6)
Laceration;	40	(12)
Vagina	23	(7)
Cervix	17	(5)
Uterine rupture	20	(6)
Iatrogenic during/after cesarean	11	(3)
Other	22	(7)
Unknown	9	(3)

^a^Up to three causes per case could be included

Table 4 presents the top three causes of postpartum hemorrhage requiring eight or more units of red blood cells according to mode of birth. The commonest cause during elective cesarean section was placenta previa (52%, *N* = 24/46), whereas uterine atony remained the leading cause for the other modes of delivery. Categorizing causes of PPH by number of red blood cell concentrates transfused, respectively ‘moderate’ (*N* = 193), ‘high (*N* = 89) and ‘immense’ (*N* = 39), showed no difference in prevalence of causes; uterine atony continued to be the main cause in each group.

**Table 4:** Top 3 causes categorized by mode of birth^a^

	N	(%)
Vaginal birth *(N = 131)*		
1. Uterine atony	84	(64)
2. Retained placenta	40	(31)
3. Placental remnant	20	(15)
Instrumental vaginal birth *(N = 43)*		
1. Uterine atony	26	(60)
2. Retained placenta	14	(33)
3. Placental remnant	6	(14)
Elective cesarean section *(N = 46)*		
1. Placenta previa	24	(52)
2. Uterine atony	17	(37)
3. Abnormally invasive placenta	13	(28)
Emergency cesarean section *(N = 105)*		
1. Uterine atony	51	(49)
2. Uterine rupture	13	(12)
3. Iatrogenic during/after cesarean	9	(9)
Termination of pregnancy *(n = 2)*		
1. Uterine atony	1	(50)
2. Uterine rupture	1	(50)

^a^Up to three causes could be included

### Management of postpartum hemorrhage requiring massive transfusion

Oxytocin (84%) was the most frequently administered uterotonic agent (prophylactic oxytocin excluded), followed by prostaglandin F2α (70%) and ergometrine (18%) (Table 5). No data regarding which uterotonic agent was administered as first line treatment were retrievable, but of 284 women who received oxytocin, 210 (74%) were given prostaglandin F2α thereafter. Eleven women who had uterine atony received no oxytocin. Instead, these women received prostaglandin F2α and one woman was supplemented with tranexamic acid.

**Table 5:** Distribution of obstetric interventions by cause; expressed as percentages

	Atony	Rupture	Previa	AIP^a^	Abruption	Retained	Total
	*(N = 179)*	*(N = 20)*	*(N = 37)*	*(N = 32)*	*(N = 20)*	*(N = 54)*	*(N = 327)*
Oxytocin	94	70	87	91	70	87	84
Prostaglandin F2α	87	50	54	72	55	85	70
Tranexamic acid	33	5	19	13	5	19	22
Ergometrine	23	15	14	19	20	28	18
Misoprostol	16	5	3	3	20	6	11
Removal of placenta^b^	31	15	11	41	15	100	29
Intrauterine balloon	32	10	14	28	5	19	23
Intrauterine packing	30	15	22	22	5	22	21
Intra-abdominal packing	6	30	0	3	0	4	7
Uterine artery ligation	6	5	8	3	5	4	5
Uterine artery embolization	29	10	19	19	5	22	22
Laparotomy	36	70	51	63	15	13	38
Re-laparotomy	8	30	5	0	0	6	9
B-Lynch suture	5	0	3	3	10	0	2
Hysterectomy	27	70	38	66	5	9	25

^a^Abnormally invasive placenta

^b^Included only removal of the placenta (or placental remnant) not performed during cesarean section

Laparotomy was performed following 42/174 (24%) vaginal deliveries and 82/151 (54%) cesarean sections. Re-laparotomy was necessary in 10/42 (24%) and 20/82 (24%) respectively. Of all 327 women, 83 (25%) underwent hysterectomy to control bleeding with highest rates in women who sustained uterine rupture (*N* = 14/20, 70%) or any form of abnormal placentation (*N* = 21/32, 66%).

### Outcome of women requiring massive transfusion

The median (IQR) length of hospitalization was 9 days (6–13 days; data missing for 14 women) and 227 women (69%) required ICU admission. The median (IQR) hemoglobin on the day of discharge was 10.15 g/dL (9.02–11.44 g/dL; data missing for 53 women). One-hundred-and-twenty-one (37%) women experienced some kind of morbidity, of whom 40 (33%) developed respiratory failure and 13 (11%) experienced renal insufficiency. Other complications were paralytic ileus (*N* = 11), heart failure (*N* = 7), Sheehan syndrome (*N* = 6) and cerebral venous sinus thrombosis (*N* = 2). Maternal death occurred in three women due to hypovolemic shock, ventricular fibrillation and massive pulmonary embolism; case fatality rate of postpartum hemorrhage requiring massive transfusion was 1 in 109 (0,9%).

## Discussion

Between 2004 and 2006, the incidence of postpartum hemorrhage treated with massive transfusion was notably high in the Netherlands (91 per 100,000 deliveries). This is four times the incidence reported for the United Kingdom between 2012 and 2013 (23 per 100,000 deliveries), and one-and-a-half times the incidence reported for the state of New York between 1998 and 2007 (60 per 100,000 deliveries) [12, 18]. We found that the leading cause of PPH with massive transfusion was uterine atony. One quarter of all women receiving massive transfusion underwent hysterectomy to control bleeding.

The difference in the incidence of massive transfusion due to PPH between the Netherlands and the UK is remarkable. Whereas incidence of major obstetric hemorrhage has differed between various countries as a result of varying inclusion criteria [15, 19, 20], our study applied the same inclusion criteria for massive transfusion described by Green et al. [12] The difference in incidence between the Netherlands and the state of New York is also of note, particularly since Mhyre et al. [18] used a higher threshold for the number of red blood cell concentrates transfused to define massive transfusion (≥10 units) and included both antepartum and postpartum hemorrhage.

A distinct difference between the national guidelines for the management of PPH between the Netherlands (Dutch Society of Obstetrics and Gynaecology (NVOG)) and the United Kingdom (Royal College of Obstetricians and Gynaecology (RCOG)) is that the RCOG specifically recommends that ‘surgical interventions should be initiated sooner rather than later’. Both guidelines are inconclusive concerning the administration of blood products; NVOG (see Additional file 2 for a summary of the NVOG PPH guideline represented as a chart [9]) recommends not to deviate from the local guidelines of the hospital, while RCOG states that the decision to provide blood transfusion ‘should be based on both clinical and hematological assessment’ [9, 10, 21]. Furthermore, it is noteworthy that the median estimated blood loss in the study from Green et al. was 6 L (4.5–8.0 L) versus 4.5 L (3.3–6 L) in our cohort, whilst the massive transfusion rate was four times higher in the Netherlands [12]. This may suggest that the difference in transfusion rate is due to differences in transfusion policy, which would emphasize the need for uniform guidelines [22].

During the study period, there were 358,874 deliveries in the Netherlands and 145,703 deliveries (40.6%) were under the responsibility of a primary care giver, making the risk of massive transfusion due to PPH 13 per 100.000 deliveries in midwifery care. Comparison of women requiring massive transfusion due to PPH with the general pregnant population in the Netherlands showed that women requiring massive transfusion had a multiple pregnancy in 11% of all cases versus 1.7% in the general population [16], suffered from preeclampsia in 17% of all cases versus 4% in the general population [23], had labour induced in 31% of all cases versus 12.5% in the general population [17], had a preterm delivery in 26% of all cases versus 5.8% in the general population [17] and delivered by cesarean section in 46% in all cases versus 13% in the general population [17]. These characteristics are known risk factors of PPH and highlight that the management of postpartum hemorrhage should not only be focused on treatment, but on prevention as well [24, 25].

Uterine atony was the most frequent cause of postpartum hemorrhage as is consistent with the literature [12, 26, 27]. Atony was also the commonest cause of PPH in home deliveries [28]. In elective cesarean sections the leading cause of massive transfusion due to PPH was placenta previa. Green et al. reported placenta accreta as the most frequent cause of PPH in women delivering by elective cesarean section, while Dupont et al. in France found that uterine atony remained the main cause of PPH regardless of mode of birth [12, 27]. The higher percentage of laparotomies performed after cesarean section is consistent with previous findings from the LEMMoN-cohort that the risk of postpartum laparotomy was more than 16 times higher in women who delivered by cesarean section compared to those who delivered vaginally [29].

As a last resort to arrest heavy bleeding, a quarter of all women underwent hysterectomy. This percentage is considerably lower than reported by Green et al. for the UK, where the overall rate of hysterectomy was 45%. A possible explanation for this difference could be the lower rates of previous cesarean deliveries; 66/327 (20%) in our study versus 73/181 (40%) in the [12]. Two studies showed that the risk of peripartum hysterectomy increased with the number of previous cesarean deliveries [30, 31]. Another contributing factor could be the higher rate of embolization in our study, 72/327 (22%) versus 29/181 (16%) in the UK, and thereby preventing the need for hysterectomy. Furthermore, uterine rupture or an abnormally invasive placenta had the highest rates of hysterectomy compared to other causes. This is coherent with the recommendation of the Dutch Society of Obstetrics and Gynaecology guideline that states that hysterectomy should not be postponed if the cause of hemorrhage is related to a placenta accreta or ruptured uterus [10].

The maternal mortality rate of massive transfusion due to PPH in our study was low with 0.84 deaths per 100.000 maternities. This is comparable with the maternal mortality rate of PPH in the Netherlands reported by Schutte et al. between 1993 and 2005 (0.7 deaths per 100.000 maternities) [32]. Nearly three-quarters of women who received massive transfusion were admitted to an ICU, and about one-third experienced morbidity. This high rate of morbidity is consistent with other studies [12, 26]. The rate of morbidity may be higher in low-income settings where not all treatment modalities are available or for Jehovah’s witnesses who refuse blood products [3, 15, 33].

A key strength is that our results were based on a nationwide cohort compromising all hospitals in the Netherlands with a maternity unit. Considering that PPH cases requiring massive transfusion must have been managed in one of these units, our results are population-based. Furthermore, our results are directly comparable to those of Green et al. who used the same definition for massive transfusion in their analysis of a UK cohort [12].

However, number of red blood cell concentrates as definition for massive transfusion remains an indicator with shortcomings as well, since it can be influenced by other factors, such as obstetrician’s decision-making. We also acknowledge that our data are from 2004 to 2006 and may not reflect the current situation. Since the incidence of PPH increased significantly throughout the years in many countries, but the incidence of obstetric blood transfusion in the Netherlands decreased [3], it is possible that the incidence of massive transfusion due to PPH may have reduced in recent years, but this is subject of further assessment. There may have been inclusion bias, since identification and management of cases may differ between obstetricians and hospitals. Underreporting is a concern, however, we have previously observed that there is a negative correlation between the rate of underreporting and the number of red blood cell concentrates transfused [34]. Therefore, we would expect a low rate of underreporting. The considerable number of women without a known Hb-level at discharge is likely due to missing data, as a result of the design of the LEMMoN-database that did not specifically include Hb-level at discharge.

Nevertheless, this study makes clear that the incidence of PPH requiring massive transfusion was high in the Netherlands at that time compared to other countries and further research of contemporary obstetric cohorts is needed to allow for more up to date international comparisons of rates of transfusion and hemorrhage-related morbidity. Networks such as the International Network of Obstetric Surveillance Systems (INOSS) could facilitate such studies [35].

## Conclusions

This study adds to the understanding of causes, management and outcomes of women with postpartum hemorrhage requiring massive transfusion and our results show that massive transfusion due to PPH is complicated by high rates of morbidity and a considerable risk of hysterectomy. The incidence of massive transfusion due to PPH appears higher in the Netherlands compared to the UK and the US. Increased vigilance for women at risk or in early stages of postpartum hemorrhage in the Netherlands is needed, while avoiding unnecessary over-transfusion. Specific reasons for the higher incidence will have to be studied in order to improve care accordingly. Our results show the importance of population-wide studies of severe maternal outcome in general, and those comparing rates of transfusion and outcomes for women with severe postpartum hemorrhage in particular.

## Additional files

Additional file 1: Distribution of the number of red blood cells concentrates transfused (PDF 142 kb).

Additional file 2: Summary chart of the Dutch Society of Obstetricians and Gynecology PPH guideline [9] (PDF 85 kb).

## Abbreviations

BMI: Body mass index
CBS: Statistics Netherlands
ICU: Intensive care unit
IQR: Interquartile range
LEMMoN: Landelijke studie naar Etnische determinanten van Maternale Morbiditeit in Nederland
LVR-2: National perinatal database
N/A: Data not available
NVOG: Dutch Society of Obstetrics and Gynaecology
PPH: Postpartum hemorrhage
RBCs: Red blood cell concentrates
RCOG: Royal College of Obstetricians and Gynaecology
UK: United Kingdom
