# Supplementary figures and images for: Incidence, management and outcome of women requiring massive transfusion after childbirth in the Netherlands: secondary analysis of a nationwide cohort study between 2004 and 2006
Source: BMC Pregnancy Childbirth. 2017 Jun 19;17:197. doi: 10.1186/s12884-017-1384-7 (PMC5477228; doi:10.1186/s12884-017-1384-7)

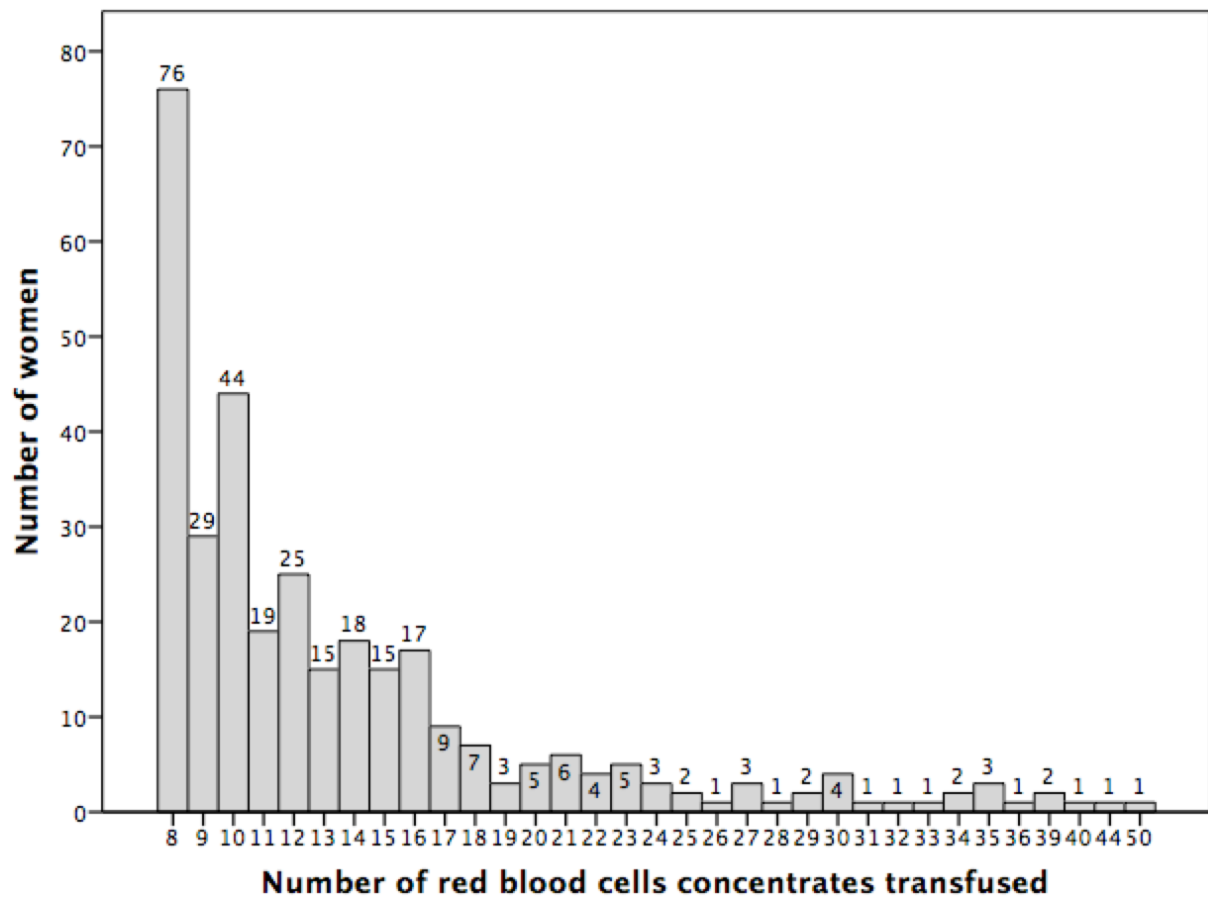

Additional file 1. Distribution of the number of red blood cells concentrates transfused.

Supplement: Supplementary file 1 — Distribution of the number of red blood cells concentrates transfused (PDF 142 kb). [file 12884_2017_1384_MOESM1_ESM.pdf]

## Guidance on treatment of postpartum hemorrhage

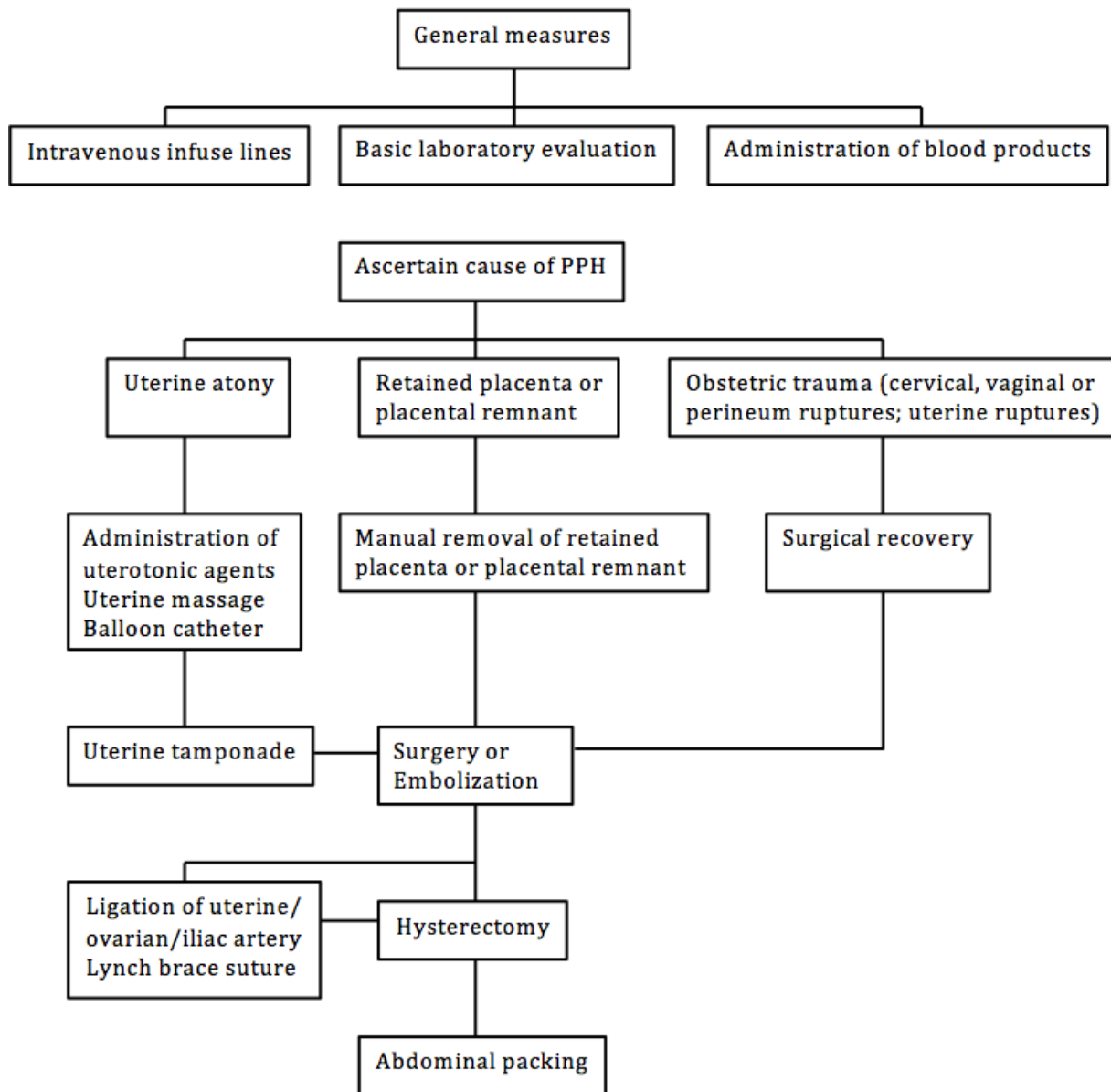

Supplement: Supplementary file 2 — Summary chart of the Dutch Society of Obstetricians and Gynecology PPH guideline [9] (PDF 85 kb). [file 12884_2017_1384_MOESM2_ESM.pdf]
